# Supplementary material for: Outbreaks of COVID-19 in Nursing Homes: A Cross-Sectional Survey of 74 Nursing Homes in a French Area
Source: J Clin Med. 2021 Sep 21;10(18):4280. doi: 10.3390/jcm10184280 (PMC8468632; doi:10.3390/jcm10184280)
Supplement: Supplementary file 1 [file jcm-10-04280-s001.zip › jcm-1326506-supplementary.pdf]

Other Supplementary materials

Size of nursing homes
Over 225 nursing homes, the median number of bed was 79 [60 to 84], comparable to that observed in our study (74 [60 to 82]).

Scores to assess the residents' level of dependency and morbidity
To assess the level of dependency and the morbidity of residents, we used different official French scales commonly used to benchmark nursing homes: the "*Groupe iso-ressources Moyen Pondéré*" (GMP), and the "*Pathos Moyen Pondéré*" (PMP) scores.
The "*Groupe iso-ressources*" (GIR) assess the level of dependency of each resident. The GIR score is ranging from 70 points (GIR-6) to 1000 points (GIR-1). An increase in points (and decrease in the GIR score) correspond to an increase in dependency. The "*Groupe iso-ressources Moyen Pondéré*" (GMP) is the average GIR of a whole facility. As for the GIR, it ranges from 70 points to 1000 points.
The "*Pathos*" assess the medical and technical care needed by a resident (*i.e.* its morbidity level), regardless of its GIR, using a model of 50 pathological states qualified by eight care profiles allowing to establish a typology of patient groups and calculate indicators of levels of care required. It ranges from 180 (low morbidity level) to 260 (high morbidity level). The "*Pathos Moyen Pondéré*" (PMP) is the average value of the residents' "*Pathos*" for a whole facility.

In the French Alps area the average GMP is ranging from 772 to 796, and the average PMP from 222 to 253. In our study, among the 74 nursing homes, the median GMP was 780 [747-819], and median PMP of 227 [206-244]

Table S1: Contingency tables used to estimate the proportion of nursing homes with COVID-19 cases among its residents and staff.

Cases among staff	Cases among residents		Total
	None	≥ 1 case	
None	26	5	32
≥ 1 case	19	24	43
Total	45	29	74

.

Table S2. COVID-19 infections, hospitalization and death among residents according to the occurrence of cases among staff and nursing home with outbreak among their residents.

Outbreak among residents	Cases among staff	Total number of residents	Confirmed or probable COVID-19		Hospitalization		Death	
		N (%)	N (%)	95CI	N (%)	95CI	N (%)	95CI
No	No	1724 (100%)	3 (0.2%)	(0.0% to 0.5%)	106 (6.1%)	(5.2% to 7.7%)	91 (5.3%)	(4.2% to 6.5%)
No	Yes	2201 (100%)	6 (0.3%)	(0.1% to 0.5%)	141 (6.4%)	(5.6% to 7.7%])	159 (7.2%)	(6.0% to 8.3%)
Outbreak	No	206 (100%)	19 (9.2%)	(4.4% to 14.3%)	18 (8.7%)	(7.7% to 19.3%])	16 (7.8%)	(3.9% to 13.4%)
Outbreak	Yes	1517 (100%)	454 (29.9%)	(27.6% to 32.3%)	323 (21.3%)	(19.3% to 23.4%])	237 (15.6%)	(13.8% to 17.5%)

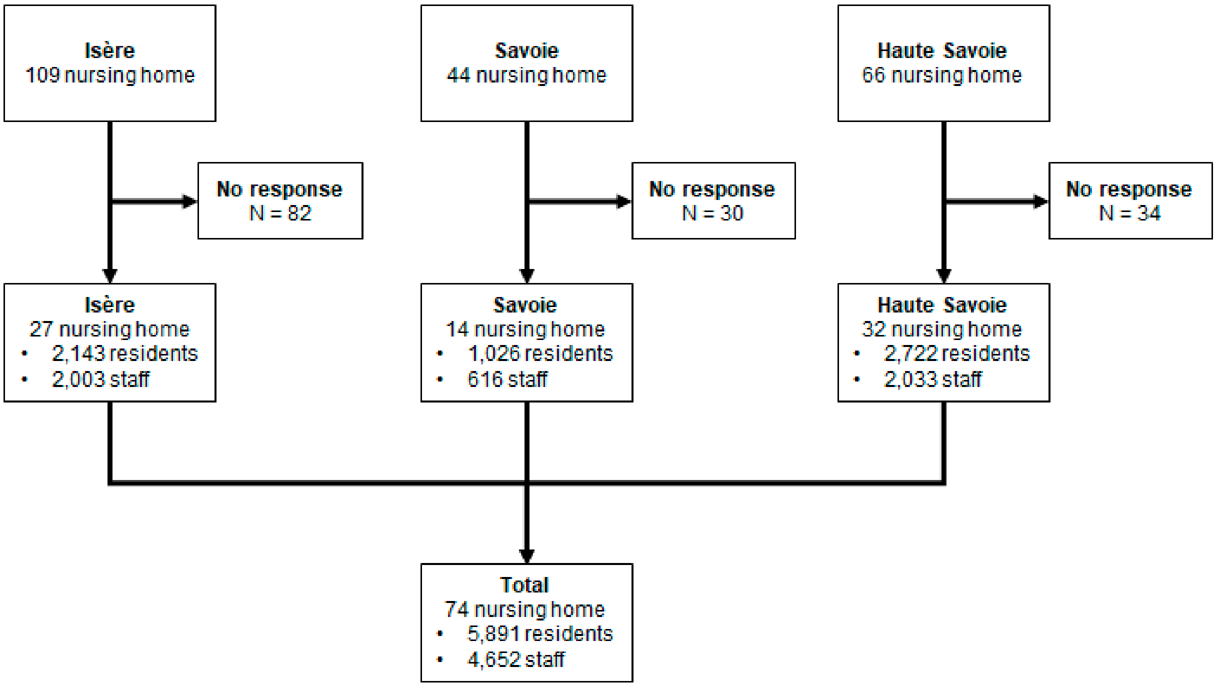

Figure S1. Flow chart of the study.

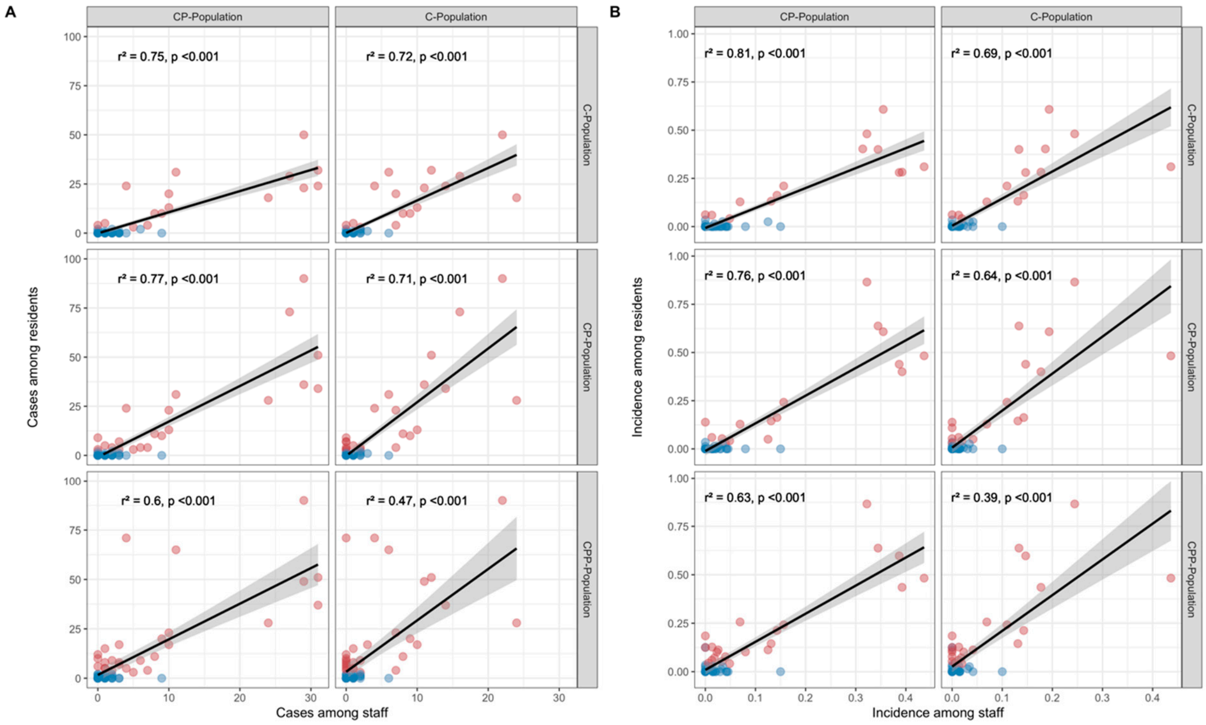

Figure S2. Linear correlations between cases and COVID-19 attack rates among staff and the residents of nursing home, by type of population: confirmed (C-Population), confirmed or probable (CP-Population), and confirmed or probable or possible (CPP-Population).

The linear coefficient of determination (r^2) between cases (panel A) and incidence (panel B) in residents and in staff are measured according to the type of population: confirmed cases (C-Population), confirmed or probable cases (CP-Population), and confirmed or probable or possible (CPP-Population) for residents, and C- and CP-Population for staff. The red dots are representing nursing homes in which outbreaks occurred, while blue dots are representing nursing homes free of outbreak. The black line represent the linear regression line between cases in residents and in personnel, and the grey area its 95% confidence interval.

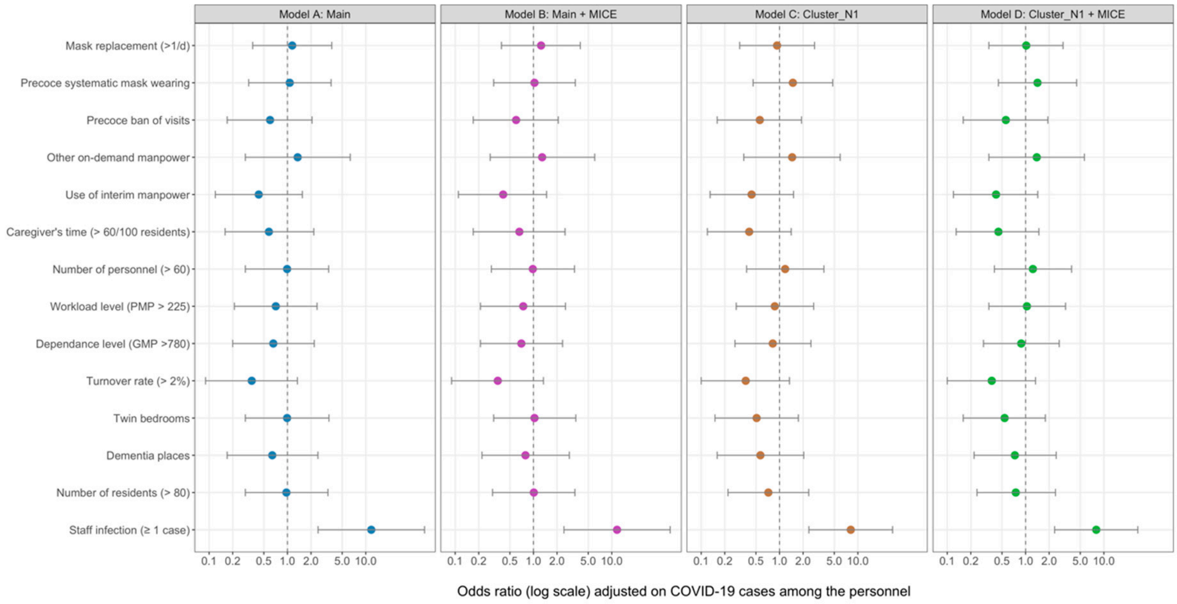

Figure S3. Variability of bivariate estimations across sensitivity analysis.

The figure represents the strength of association between nursing home characteristics and the occurrence of outbreaks (≥3 cases) among the residents, adjusted on the occurrence of cases ≥1 among the personnel. The results of the main analysis (Model A) are compared to the three sensitivity analysis (Model B, Model C, and Model D). The associations were analysed using logistic regression and are reported as odds ratio (OR) and their 95%CI. A logarithmic scale is used to avoid misinterpretation in estimates, as when the coefficients (and their associated intervals) are exponentiated to compute OR, they are no longer symmetric on a linear scale.
